# Responsiveness of afferent renal nerve units in renovascular hypertension in rats

**DOI:** 10.1007/s00424-021-02591-6

**Published:** 2021-07-07

**Authors:** Kristina Rodionova, Karl F. Hilgers, Salman Rafii-Tabrizi, Johannes Doellner, Nada Cordasic, Peter Linz, Anna-Lena Karl, Christian Ott, Roland E. Schmieder, Mario Schiffer, Kerstin Amann, Roland Veelken, Tilmann Ditting

**Affiliations:** 1 Department of Internal Medicine 4 - Nephrology and Hypertension, Friedrich-Alexander University Erlangen, Erlangen, Germany; 2 Department of Radiology, Friedrich-Alexander University Erlangen, Erlangen, Germany; 3 Department of Internal Medicine 4 – Nephrology and Hypertension, Paracelsus Private Medical School Nuremberg, Nuremberg, Germany; 4 Department of Nephropathology, Friedrich-Alexander University Erlangen, Erlangen, Germany

**Keywords:** Hypertension, Renal innervation, Neuronal cell culture, Renovascular hypertension, Mesangioproliferative Glomerulonephritis, Renal denervation

## Abstract

Previous data suggest that renal afferent nerve activity is increased in hypertension exerting sympathoexcitatory effects. Hence, we wanted to test the hypothesis that in renovascular hypertension, the activity of dorsal root ganglion (DRG) neurons with afferent projections from the kidneys is augmented depending on the degree of intrarenal inflammation. For comparison, a nonhypertensive model of mesangioproliferative nephritis was investigated. Renovascular hypertension (2-kidney, 1-clip [2K1C]) was induced by unilateral clipping of the left renal artery and mesangioproliferative glomerulonephritis (anti-Thy1.1) by IV injection of a 1.75-mg/kg BW OX-7 antibody. Neuronal labeling (dicarbocyanine dye [DiI]) in all rats allowed identification of renal afferent dorsal root ganglion (DRG) neurons. A current clamp was used to characterize neurons as tonic (sustained action potential [AP] firing) or phasic (1–4 AP) upon stimulation by current injection. All kidneys were investigated using standard morphological techniques. DRG neurons exhibited less often tonic response if in vivo axonal input from clipped kidneys was received (30.4% vs. 61.2% control, *p* < 0.05). However, if the nerves to the left clipped kidneys were cut 7 days prior to investigation, the number of tonic renal neurons completely recovered to well above control levels. Interestingly, electrophysiological properties of neurons that had in vivo axons from the right non-clipped kidneys were not distinguishable from controls. Renal DRG neurons from nephritic rats also showed less often tonic activity upon current injection (43.4% vs. 64.8% control, *p* < 0.05). Putative sympathoexcitatory and impaired sympathoinhibitory renal afferent nerve fibers probably contribute to increased sympathetic activity in 2K1C hypertension.

**Supplementary Information:**

The online version contains supplementary material available at 10.1007/s00424-021-02591-6.

## Introduction

After high hopes were raised with respect to the potential of renal denervation in the treatment of arterial hypertension [29], the sobering results of the Simplicity 3 Trial in 2015 [5] questioned the future of this innovative procedure in the treatment of high blood pressure (BP). In the meantime, new and carefully designed studies with specific questions in well-controlled cohorts [1, 14, 47] suggest far smaller BP effects of renal denervation than originally assumed, that maybe nevertheless of therapeutic significance for the treatment of resistant hypertension [37].

Regardless of these new developments in the field of renal nerve ablation, it is still controversial how renal innervation influences systemic autonomous nerve activity [37]. Numerous publications favor the role of renal afferent innervation [2–4, 8, 33, 36]. In patients with end-stage renal disease and/or hypertension that have undergone bilateral nephrectomy [6, 19], normalization of formerly increased sympathetic activity as assessed by recordings of peripheral sympathetic nerve fibers strongly suggests an important role of renal sensory afferent innervation in the regulation of efferent sympathetic tone. Unfortunately, these clinical observations did not answer the question of how afferent renal nerve pathways will influence sympathetic activity in hypertension.

Decreases in peripheral sympathetic activity after nephrectomy support the concept of renal sympathoexcitatory afferents [50]. More recent studies in hypertension models with distinguishable characteristics clearly favor sympathoexcitatory effects [2–4, 33, 36].

Since single afferent renal neurons are difficult to investigate in vivo, we have developed an in vitro model of renal afferent innervation, allowing for the investigation of dorsal root ganglion (DRG) neurons with former renal afferent nerve projections in vivo [12, 16]. Using this model, we could demonstrate earlier that renal DRG neurons comprise a much higher proportion of neurons that respond with a high frequency of action potential (AP) due to depolarizing current injection, as compared to neurons with axons from other sites [12, 16]. DRG neurons were categorized by their firing pattern as “tonic” or “phasic” [12, 44]. Neurons that show sustained AP generation during depolarizing current injection were defined as “tonic.” In contrast, neurons showing only transient AP generation during current injection were defined as “phasic.”

We could earlier demonstrate that a low amount of neurons with a tonic response pattern upon stimulation is correlated with decreased afferent nerve activity [42]. This finding suggests that higher afferent renal nerve activity is dependent on a respective higher number of tonically responding neurons with renal afferents [10, 42]. Furthermore, in a recent study in rats with renovascular hypertension, increased afferent renal nerve activity occurred with increased sympathetic nerve activity [33]: the interpretation of this finding leads to the assumption that afferent renal nerve activity is stimulating central sympathetic output under these circumstances.

Data of ours also suggested that inflammation and fibrosis influence afferent nerve pathways of the kidney [9, 41].

These findings made us test the hypothesis that in experimental renovascular hypertension (2-kidney, 1-clip [2K1C]), the excitability of afferent renal pathways is increased as assessed by the gain of tonic, potentially highly active neurons in respective neuronal cell culture samples dependent on the degree of renal inflammation and fibrosis in the respective animals.

Alterations in arterial hypertension are confounding variables influencing the function of the autonomous nervous system, e.g., the genuine central generation of sympathetic outflow is augmented [46], and increased peripheral sympathetic nerve activity influences the neurophysiology of afferent nerve pathways [24]. Therefore, we introduced a normotonic model of mesangioproliferative nephritis for comparison that we had investigated intensively over the last years concerning the function of renal innervation in inflammation and fibrosis [41, 42, 49].

We used our cell culture model for the investigation of DRG neurons with afferent axons from the kidneys. We harvested T11-L2 DRG neurons from animals with renovascular hypertension (2-kidney, 1 clip [2K1C]). In these rats, the left kidney is exposed to high and the right kidney to low BP with putatively different degrees of renal inflammation. We also investigated DRG neuron samples from denervated and clipped left kidneys from 2K1C animals since renal denervation in animal models of renovascular hypertension suggests involvement of renal nerves in the pathophysiology of this form of secondary hypertension [30, 31]. Even selective afferent denervation alone had a significant influence on experimental renovascular hypertension [33, 36].

## Material and methods

### Animals

Male Sprague–Dawley (SD) rats (Charles River, Sulzfeld, Germany) weighing 180–250 g (9 to 12 weeks of age) were maintained in our animal facility at 24 ± 2 °C. They were fed a standard rat diet (no. C-1000, Altromin, Lage, Germany) containing 0.2% sodium by weight and had free access to tap water. All procedures performed in animals were done in accordance with the guidelines of the American Physiological Society and in compliance with the NIH Guide for Animal Care and Use in Laboratory Practice.

### Anesthesia

For all procedures, anesthesia was induced with a mixture of O_2_, 50% N_2_O, and 5% of isoflurane (Isofuran CP® 1 ml/ml, CP Pharma, Burgdorf, Germany), while the latter was rapidly switched to a maintenance dose of ~ 1.5%. Additional analgesia was ensured by a single dose of meloxicam (1 mg/kg) given subcutaneously.

### Disease models

The 2K1C model of *renovascular hypertension* was induced as described previously by placing a silver clip of a 0.2-mm ID around the left renal artery through a flank incision under anesthesia [10, 18]. Control animals underwent a sham procedure. The animals were followed by daily measurements of weight and weekly measurements of systolic blood pressure (BP) by tail-cuff plethysmography in anesthesia. The identities of animals with renovascular hypertension and sham-operated animals were blinded to the observer. Animals were only included in the 2K1C group if systolic BP was > 150 mmHg. Animals that failed to thrive or lost weight were excluded after 2 weeks. After 3 weeks, renal neurons were harvested and kidneys prepared for further investigations.

In one subgroup of 2K1C rats, the clipped kidney was denervated 1 week before tissue removal, i.e., 2 weeks after clipping, during the same procedure when the dicarbocyanine dye (DiI) was applied to label the DRG neurons with renal afferents (details as follows). A left flank incision was made and then the renal denervation was performed by surgically stripping the left clipped renal artery and vein of the adventitia and cutting all renal nerve bundles visible under a dissection microscope (× 25 to × 40). After that, a tiny drop of DiI was applied to the proximal end of the cut renal nerves. Thereafter, the wound was closed in layers [39].

### Mesangioproliferative glomerulonephritis

Mesangioproliferative glomerulonephritis (GN; anti-Thy1.1 nephritis) was induced by a single intravenous injection of a 1.75-mg/kg OX-7 monoclonal antibody (mAb) into the tail vein [49] or respective sham treatment. Nephritic rats were kept in metabolic cages to determine urinary albumin excretion in 24 h. The rat albumin ELISA quantitation kit (Bethyl Laboratories, Montgomery, USA) was set up using Nunc-Immuno 96-well flat-bottom high-binding MaxiSorp polystyrene microtiter plates (Nunc, Roskilde, Denmark) to quantitatively measure levels of urinary albumin [49]. Only rats exhibiting marked increases of urinary albumin were regarded as animals with successful GN induction [49]. After 1 week, renal neurons were harvested and kidneys prepared for further investigations.

### Labeling of DRG neurons with renal afferents

To identify DRG neurons that receive projections from the kidney, the dicarbocyanine dye (DiI, 1,1′dioleyl-3,3,3′ tetramethyl-indocarbocyanine methanesulfonate, in EtOH; Molecular Probes™, Fischer Scientific, Schwerte, Germany) was applied to both lower renal poles by subcapsular application (5 μl of a solution of 10 g DiI/L) in anesthetized rats, as described previously [9, 16, 42]. In 2K1C rats, labeling was done 2 weeks after left renal artery clipping; in anti-Thy1.1 rats, labeling was done on the same day when nephritis was induced. In the denervated subgroup of 2K1C rats, 2 µl (10 g DiI/L) of DiI were applied to the renal nerves only on the left side 14 days after clipping by the use of a heat-pulled borosilicate glass pipette, but no bilateral subcapsular DiI application was performed in this subgroup. DiI remained on the nerves for at least 3 min in order to allow penetration of the intact cytoplasmatic membranes of the dendrites. DiI inserts into the membranes rapidly and is transported to the somata by axoplasmatic transport [21–23, 43]. Afterwards, all nerves of the left kidney were destroyed.

### Blood pressure measurement and tissue sampling

A left-sided arterial femoral catheter was implanted and connected to a transducer (Statham P23Db) to record arterial blood pressure (mean arterial pressure) and heart rate (HR) (pressure processor type 13–4615-52; Gould Instrument Systems, Valley View, OH) [10].

After BP measurements, all rats were sacrificed; DRG (T11-L2) harvested; and finally, kidneys excised, weighed, and decapsulated. Renal tissue was fixed in methyl-Carnoy (MC) solution (60% methanol, 30% chloroform, and 10% glacial acetic acid) for later histology and immunohistochemistry [49].

### Neuronal cell culture

Seven days after each labeling procedure in the different groups, the rats were again anesthetized, and DRGs from T11-L2 were collected. Neurons were isolated by mechanical and enzymatic dissociation as described previously [9, 16, 42]. The ganglia were incubated with collagenase IA (2 mg/ml C9891, Sigma-Aldrich, Munich, Germany in DMEM, PAA Laboratories GmbH, Linz, Austria) for 1 h in 5% CO_2_ at 37 °C. Enzymatic dissociation was terminated by replacing collagenase-containing DMEM with fresh DMEM + cultural medium (DMEM + , i.e., DMEM plus 10% FCS, 1% penicillin/streptomycin, and 0.1% insulin). Tissue digestion was stopped by FCS. The ganglia were triturated using sterile Pasteur pipettes (Sigmacote®; Sigma-Aldrich, Munich, Germany) to dissociate individual cells. After centrifugation at 100 relative centrifugal force, cells were resuspended in 10 ml DMEM + and centrifuged once more. The pellet was resuspended in 1.8 ml DMEM + , and cells were plated on glass coverslips coated with poly-L-lysine. The coverslips with neurons were cultured in DMEM + for one day before electrophysiological experiments.

### Patch-clamp experiments: influence of various disease models (2K1C, anti-Thy1.1) on electrophysiological properties of cultivated renal afferent DRG neurons

Patch-clamp recordings were made within 30 h of plating. The largest group of cells was represented by medium-capacitance and medium-size neurons as previously described [12].

Patch-clamp recordings were obtained using a pipette solution containing 140 mM KCl, 5 mM NaCl, 2 mM MgCl_2_, 1 mM CaCl_2_, 2 mM Mg-ATP, 0.3 mM Na-GTP, 10 mM EGTA. and 10 mM HEPES, pH 7.4. Recordings were conducted in whole-cell mode using pipette resistances of 2–4 MΩ. Patch-clamp recordings were obtained with an Axopatch 200B amplifier (Axon Instruments, Foster City, CA). Data were sampled at 5 kHz for voltage-clamp or 20 kHz for current-clamp measurements and analyzed with pClamp® 10.2 (Axon Instruments, Foster City, CA). For current-clamp measurements, an extracellular solution of 140 mM NaCl, 5 mM KCl, 1 mM MgCl_2_, 10 mM HEPES, and 10 mM glucose was used.

Only neurons with a resting membrane potential below − 40 mV were measured. Cells that stained brightly for DiI to laser excitation (540 nm) were considered renal afferent neurons. Nonrenal neurons from the same cultures that showed no DiI staining were not tested. All recordings were done at room temperature, i.e., 22 ± 2 °C.

Cell capacitance was compensated manually, and cell parameters (size, capacitance, and resistance) were documented. To confirm the vitality of each neuron, typical neuronal currents were examined using a voltage step protocol (step duration of 50 ms, from − 100 to + 60 mV in 17 steps with a 1-s delay). Neurons were identified by the presence of fast sodium currents during depolarization.

### Current-clamp protocols

To determine DRG firing patterns (tonic vs. phasic), we utilized a current-clamp approach as recently described [12, 42, 44]. PClamp® 10.2 (Axon Instruments, Foster City, CA) was used to control current-pulse generation, to record membrane potentials, and for offline data analysis. Action potentials (APs) were induced by rectangular current-pulse injections as follows: a 5-ms pre-pulse, followed by a 600-ms pulse with an interpulse delay of 100 ms, was delivered in three consecutive trains of increased intensity (40–400, 400–4.000, 4000–12.000 pA), in 10 consecutive steps (5.16 s, each). We categorized DRG neurons as “tonic” or “phasic” as described previously [12, 44]. In short, neurons exhibiting 1–4 APs upon suprathreshold current injection were regarded as “phasic,” and neurons exhibiting > 4 APs as “tonic.”

### Kidney histology

For evaluation of histology, renal tissue of all experimental animals and respective controls was collected, fixed overnight in MC solution, dehydrated in increasing concentrations of methanol, followed by 100% isopropanol, embedded in paraffin, and cut in a standardized manner with periodic acid–Schiff (PAS) [49]. Four-micrometer paraffin sections were cut and stained. The evaluation of kidney histology and immunohistochemistry was carried out on representative sections in a blinded manner.

### Immunohistochemistry

After deparaffinization, peroxidase activity was blocked with 3% H_2_O_2_ for 20 min. A mouse mAb against the macrophage marker ED1 (Serotec, Biozol, Eching, Germany) and a goat polyclonal antibody to collagen IV (Southern Biotechnology Associates, Birmingham, AL, USA) were used in respective dilutions. Immunostaining was carried out with a 0.1% diaminobenzidine tetrahydrochloride/0.02% H_2_O_2_ detection system (Vector Laboratories, Burlingame, CA). Intraglomerular ED1 positive cells were counted as positive cells per glomerular area in all glomeruli in representative and comparable sections of each kidney (120–300 glomeruli) and expressed as cells per glomerular section. Interstitial ED1 positive cells were counted in 20 medium-power (magnification × 250) cortical views per section and expressed as cells per mm^2^ of interstitional area. Glomerular collagen IV staining was measured by Metaview (Visitron Systems, Puchheim, Germany) in every third glomerulus per cross section, and the stained area was expressed as a percentage of the total area of the glomerular tuft as performed many times before [49]. Moreover, the evaluation of kidney histology and immunohistochemistry was carried out in a blinded manner.

### Data analysis

All data underwent normality testing (Shapiro–Wilk) to assess the distribution of data; *t*-tests were applied for comparison of two groups with normal distribution; otherwise, nonparametric testing (Mann–Whitney rank-sum test) was used. More than two groups were evaluated by one-way ANOVA with Ducan’s post hoc test with normally distributed data; otherwise, Kruskal–Wallis one-way ANOVA of ranks was selected with Dunn’s post hoc method.

Statistical significance was defined as *p* < 0.05 (two sided). The *z*-test was used to test for significant differences in the frequency distribution of characteristics of DRG neurons (e.g., tonic vs. phasic; anti-Thy1.1 vs. control; control vs. clipped vs. clipped denervated vs. non-clipped; all pairwise). Data are presented as group means ± SD or as box-whisker plots, in which the box boundaries denoted the first and third quartiles and whiskers indicated the 5th and 95th percentiles. SigmaPlot 14 and SigmaStat 3.5 (Systat Software, Erkrath, Germany) were selected for statistical analysis and graphical display.

## Results

### Baseline parameters

Baseline data for rats with renovascular hypertension or mesangioproliferative GN are summarized in Table 1. Briefly, mean arterial BP was significantly higher in 2K1C than in controls, whereas body weight was significantly lower and heart rate was comparable between both groups (Table 1). In anti-Thy1.1, nephritis albuminuria was significantly higher than in controls, whereas BP, body weight, and heart rate were comparable (Table 2).

**Table 1:** Baseline parameters of rats with renovascular hypertension (2K1C)

	Renovascular hypertension		Sham-operated control (*n* = 12)
	Intact innervation (*n* = 18)	Clipped kidney denervated (*n* = 13)	
Mean arterial blood pressure (mmHg)	188.3 SD (10.3)* *p < 0.001 Normality test failed	169.1 SD (31.6)* *p = 0.004 Normality test failed	106.5 SD (7.4)
Heart rate (beats/min)	384 SD (25)	376 SD (22)	368 SD (19)
Body weight (g)	286.3 SD (11.4)* *p < 0.001 Normality test failed	276.7 SD (34.8)* *p < 0.001 Normality test failed	318.8 SD (12.6)
Heart weight (g)	4.65 SD (0.32)* *p < 0.0001 Normality test failed	4.35 SD (0.76)* *p < 0.0001 Normality test failed	3.16 SD (0.32)
Clipped kidney/body weight ratio (g/kg)	3.8 SD (0.9)	4.3 SD (1)	-
Non-clipped kidney/body weight ratio(g/kg)	6.2 SD (0.7)* *p < 0.001 Normality test failed	6.3 SD (1.2) *p < 0.001 Normality test failed	-
Sham kidney/body weight ratio (g/kg)	-	-	4.0 SD (0.5)

^*^Renovascular vs. sham-operated control; means + SD; normality test failed: ANOVA on ranks and Dunn’s method; normality test passed: ANOVA and Duncan’s post hoc test

**Table 2:** Baseline parameters of rats with mesangioproliferative glomerulonephritis (anti-Thy1.1)

	mesangioproliferative glomerulonephritis (n = 10)	Control (n = 8)
mean arterial blood pressure *(mm Hg)*	104.3 SD (9.5)	107.4 SD (6.3)
heart rate *(beats/min)*	373 SD (29)	360 SD (22)
body weight *(g)*	315 SD (12)	320.8 SD (10)
kidney/body wt ratio *(g/kg)*	4.1 SD (0.6)	3.9 SD (0.4)-
Albuminuria *(ug/24 h)*	145 SD (28)	3.2 SD (1.8)

^*^mesangioprolif. *vs.* control; means + SD; Normality test passed: t test

### *Neurons with renal axons — electrophysiological assessment *in vitro

Cultured DRG neurons could be easily distinguished from fibroblasts and other cells by their typical size and morphology. About one quarter of these neurons was brightly labeled with DiI. Only these neurons were regarded as renal afferent neurons and used for further investigations [9, 16, 42]. Unlabeled, i.e., nonrenal neurons, were not investigated in our study.

**Fig. 1:**
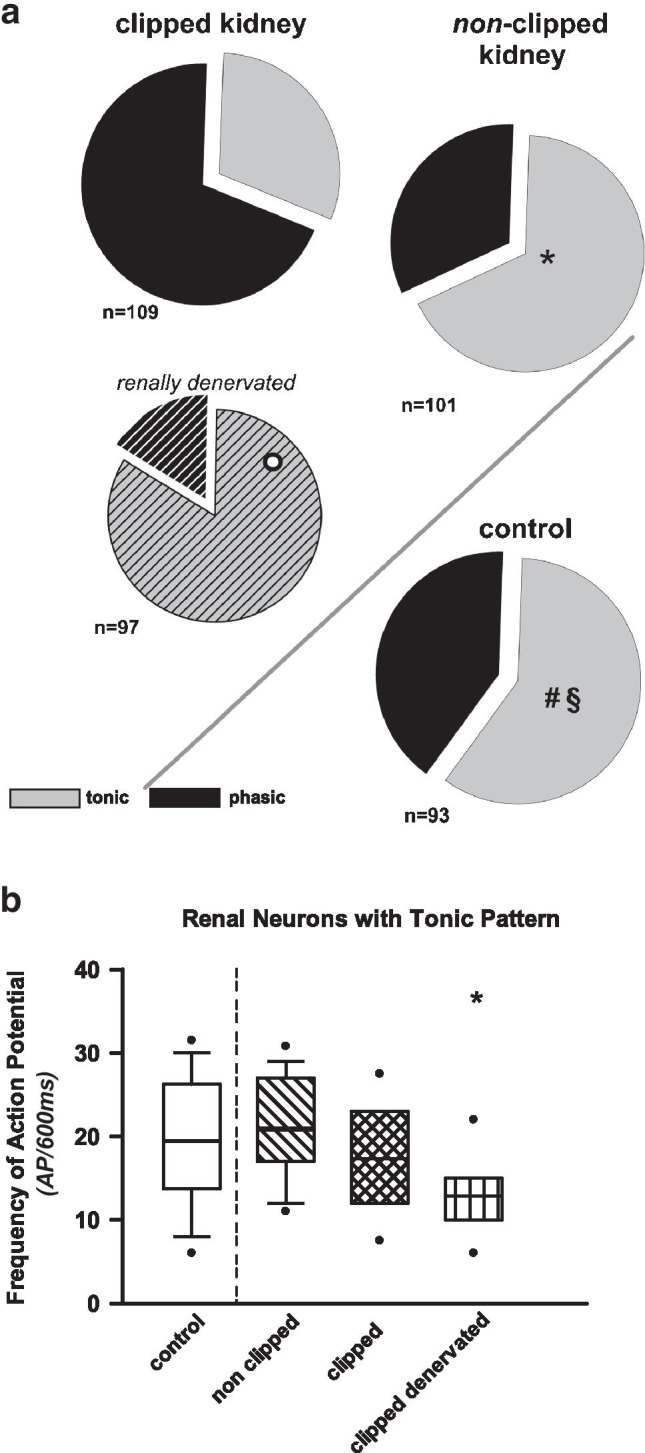
**a** Relative proportion of tonic (highly active) and phasic (more silent) afferent DRG neurons in samples from clipped and non-clipped kidneys of hypertensive 2K1C rats and normotensive controls. Proportions of tonic neurons from non-clipped hypertensive 2K1C vs. normotensive control kidneys were not different. However, neurons with axons from the clipped kidneys exhibited a significantly lower portion of tonic neurons. This distribution was significantly altered when clipped 2K1C kidneys were denervated 7 days before harvesting the neurons for cell culture: the number of tonic neurons increased significantly. *p < 0.001 (z-test) tonic clipped vs. tonic non-clipped; #*p* < 0.001 (z-test) tonic control vs. tonic clipped; *p* < 0.001 (z-test) tonic clipped vs. tonic clipped denervated; §*p* = 0.638 (z-test) tonic control vs. tonic non-clipped. **b** Frequency of APs of renal tonic neurons from normotensive controls and non-clipped, clipped, and clipped denervated 2K1C rats. Frequency of APs from clipped denervated kidneys was significantly lower as compared to control (clipped denervated vs. all other groups, *p = 0.010, normality test failed, ANOVA of ranks, all pairwise)

Current-clamp experiments were performed to classify the neurons into “phasic” (i.e., 1–4) APs and “tonic” (i.e., > 4 APs) [12, 44] according to their firing pattern due to suprathreshold current injection. The following neuron sample groups were investigated: *renovascular hypertension* (normotensive control sample: *n* = 93; 2K1C-hypertensive sample, non-clipped kidney: *n* = 101; 2K1C-hypertensive sample, clipped kidney: *n* = 109; 2K1C-hypertensive sample, clipped kidney, denervated: *n* = 97) and *mesangioproliferative nephritis* (control sample: *n* = 61; nephritic sample: *n* = 55). In renal afferent DRG neuron samples from rats with renovascular hypertension, the fraction of tonic neurons was significantly lower with respect to the clipped kidney. However, with respect to the non-clipped kidney, the distribution of tonic to phasic neurons was almost the same as in renal neurons of normotensive control animals (Fig. 1; Supplementary material: Figures S1, S2).

Interestingly, when the clipped kidneys were denervated 7 days prior to the harvest of DRG neurons, the proportion of tonic (potentially highly active) neurons had significantly increased as compared to clipped kidney DRG neurons from 2K1C rats without renal denervation (Fig. 1a). However, the AP frequency had not reached the level seen in controls (Fig. 1b). Of note, the denervation of the clipped kidney did not only influence the frequency of APs but also increased the action potential duration significantly as compared to all other tonic groups of neurons (see Table 3). Furthermore, the threshold voltage after denervation was less negative. Significant differences were also observed between the tonic and phasic neurons (see Table 3). The blood pressure tended to be lower already.

**Table 3:**
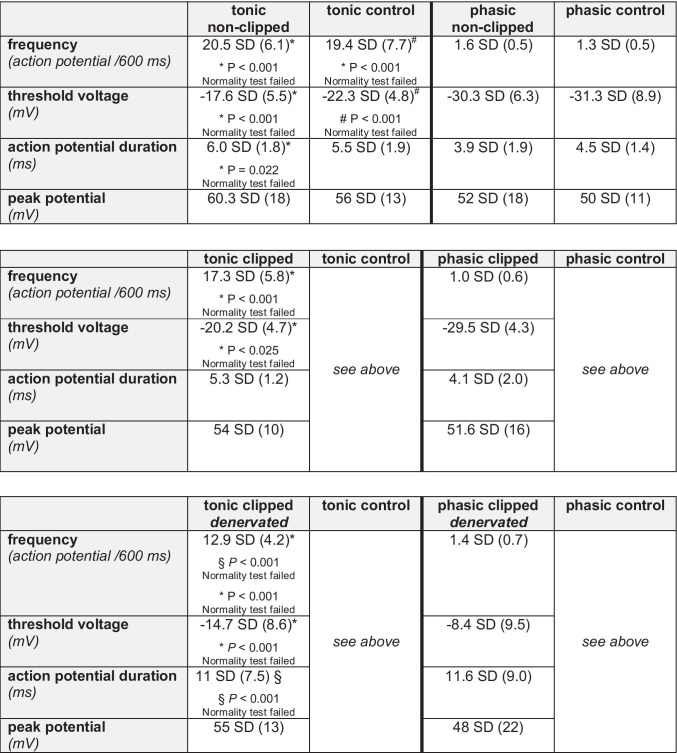
Electrophysiological characteristics of dorsal root ganglion (DRG) neurons with afferent axons from the kidneys in renovascular hypertension (2K1C); (mean [SD]).

*Tonic vs. phasic; #tonic control vs. phasic control; §tonic clipped denervated vs. all other tonic groups Data are displayed as means ± SD. Normality test (Shapiro–Wilk) passed: one-way ANOVA with Ducan’s post hoc test

Normality test (Shapiro–Wilk) failed: Kruskal–Wallis one-way ANOVA of ranks and Dunn’s post hoc test

**Fig. 2:**
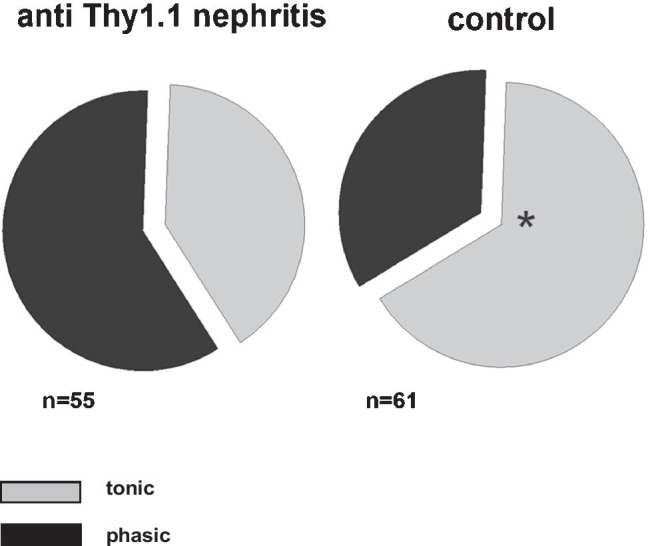
Relative proportion of tonic and phasic neurons in samples from rats with mesangioproliferative (anti-Thy1.1) nephritis and controls. In samples of renal neurons from nephritic rats, a significantly decreased number of neurons with tonic response pattern was found (*p < 0.001 (z-test) tonic control vs. tonic nephritic)

In renal samples from rats with mesangioproliferative GN, the number of tonic neurons with renal afferents was significantly decreased (Fig. 2; Supplementary material: Figure S3).

Renal neurons from rats with renovascular hypertension and nephritic rats and controls were further analyzed according to their electrophysiological properties, i.e., capacity, membrane resistance, and resting potential. As expected, no differences were found in these parameters. Experimental nephritis did only change the tonic-to-phasic ratio, but also did not influence other electrophysiological parameters as compared to controls. Significant differences in these parameters were only observed between the tonic and phasic groups (see Tables 3 and 4).

**Table 4:** Electrophysiological characteristics of neurons from the dorsal root ganglion with afferent axons from the kidneys in anti-Thy1.1 nephritic rats [mean (SD)]. Anti-Thy1.1 nephritis (nephritic *n* = 61; control *n* = 55)

	Tonic nephritic	Tonic control	Phasic nephritic	Phasic control
Frequency (action potential/600 ms)	19.1 SD (5.8)* *p < 0.001 Normality test failed	18.0 SD (3.4)# *p < 0.001 Normality test failed	1.5 SD (0.7)	1.5 SD (0.7)
Threshold voltage (mV)	− 20.2 SD (5.0)* *p < 0.001 Normality test failed	− 19.6 SD (4.3)# *p < 0.001 Normality test failed	− 29.3 SD (6.2)	− 27.4 SD (5.8)
Action potential duration (ms)	6.4 SD (2.1)	5.5 SD (1.9)	4.3 SD (2.0)	4.9 SD (2.3)
Peak potential (mV)	58.3 SD (17)* *p < 0.001 Normality test failed	55.3 SD (13)	38.9 SD (12.4)	45.9 SD (15.4)

^*^Tonic nephritic vs. phasic nephritic; #tonic control vs. phasic control; data are displayed as means + SD; normality test passed: *t*-test; normality test failed: Mann–Whitney rank-sum test. Experimental nephritis did not influence the displayed parameters with respect to the tonic or phasic groups of renal neurons as compared to controls. Significant differences were only observed between the tonic and phasic groups

### Renal morphology in renovascular hypertension and mesangioproliferative glomerulonephritis

**Fig. 3:**
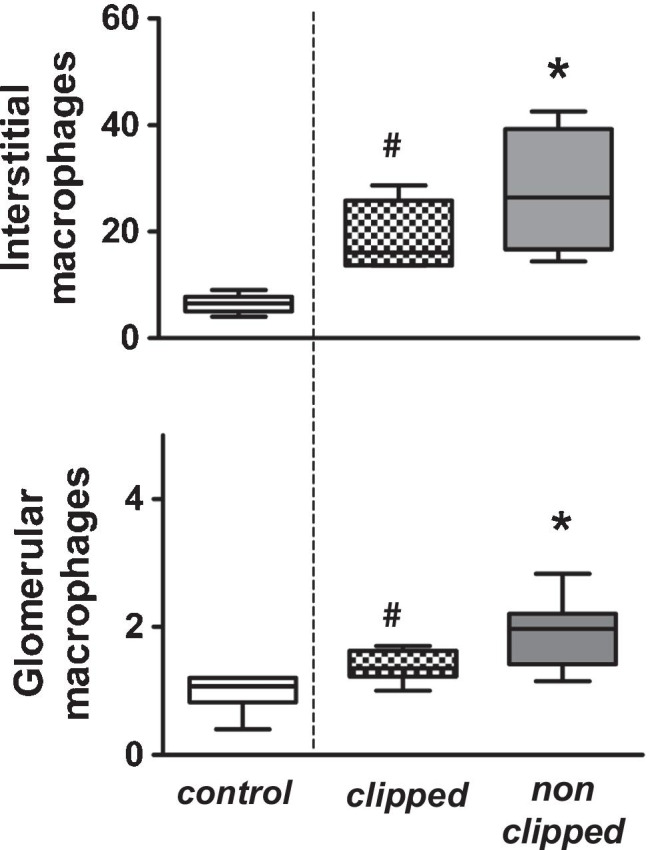
Alteration macrophages in the clipped and non-clipped kidneys of 2K1C rats and controls determined by immuno-histochemistry. Interstitials (upper panel) and glomerular macrophages (lower panel) were significantly increased in the kidneys from renovascular hypertension as compared to controls. *n* = 6, (*p < 0.05). Upper panel: interstitial macrophages (in cells/glomerular section; control vs. clipped (#) vs. non- clipped (*). *n* = 6 per group, #*p* = 0.016 and *p = 0.001; normality test failed, ANOVA of ranks, all pairwise). Lower panel: glomerular macrophages (in cells/glomerular section, control vs. clipped (#) vs. non- clipped (*). *n* = 6 per group, #*p* = 0.002 and *p = 0.039, normality test passed, one-way ANOVA, all pairwise)

Twenty-one days after induction of renovascular hypertension, significant increases of glomerular and interstitial ED1 positive cells were observed in the clipped and non-clipped kidneys (Fig. 3; Supplementary material: Figure S4). This finding tended to be more pronounced in the non-clipped kidney, but no statistically significant findings between clipped and non-clipped kidney were observed.

**Fig. 4:**
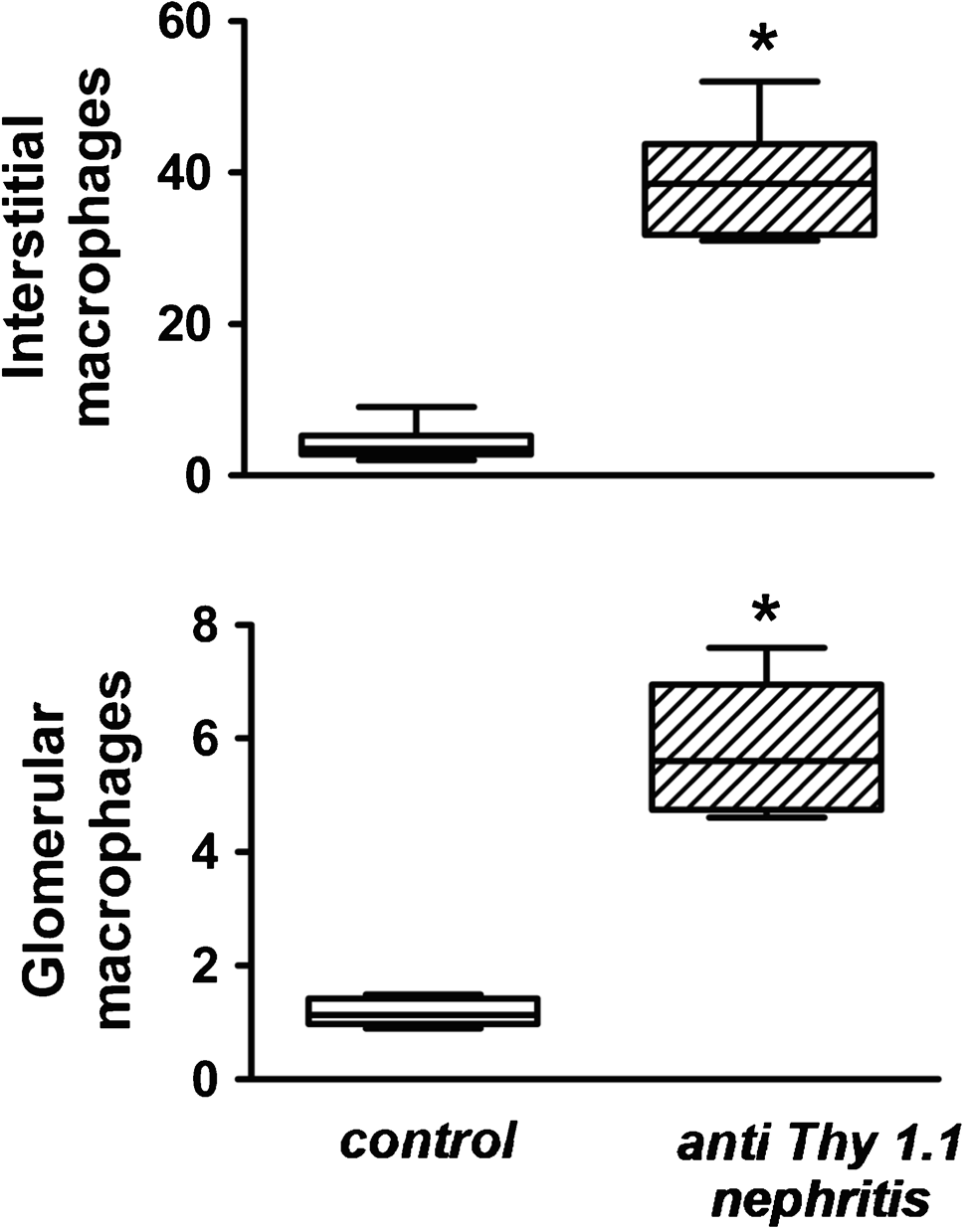
Alterations of renal macrophages in mesangioproliferative (anti-Thy1.1) nephritis and controls determined by immunohistochemistry. Interstitial (upper panel) and glomerular macrophages (lower panel) were significantly increased in nephritic kidneys as compared to controls. Upper panel: interstitial macrophages (in cells/glomerular section, control vs. anti-Thy1.1 (*). *n* = 6 per group, *p = 0.001, normality test passed, one-way ANOVA, all pairwise). Lower panel: glomerular macrophages (in cells/glomerular section, control vs. anti-Thy1.1 (*). *n* = 6 per group, *p = 0.004, normality test failed, ANOVA of ranks, all pairwise)

In rats with mesangioproliferative GN, glomerular and interstitial ED1 positive cells were increased in nephritic rats as compared to controls (Fig. 4; Supplementary material: Figure S5).

**Fig. 5:**
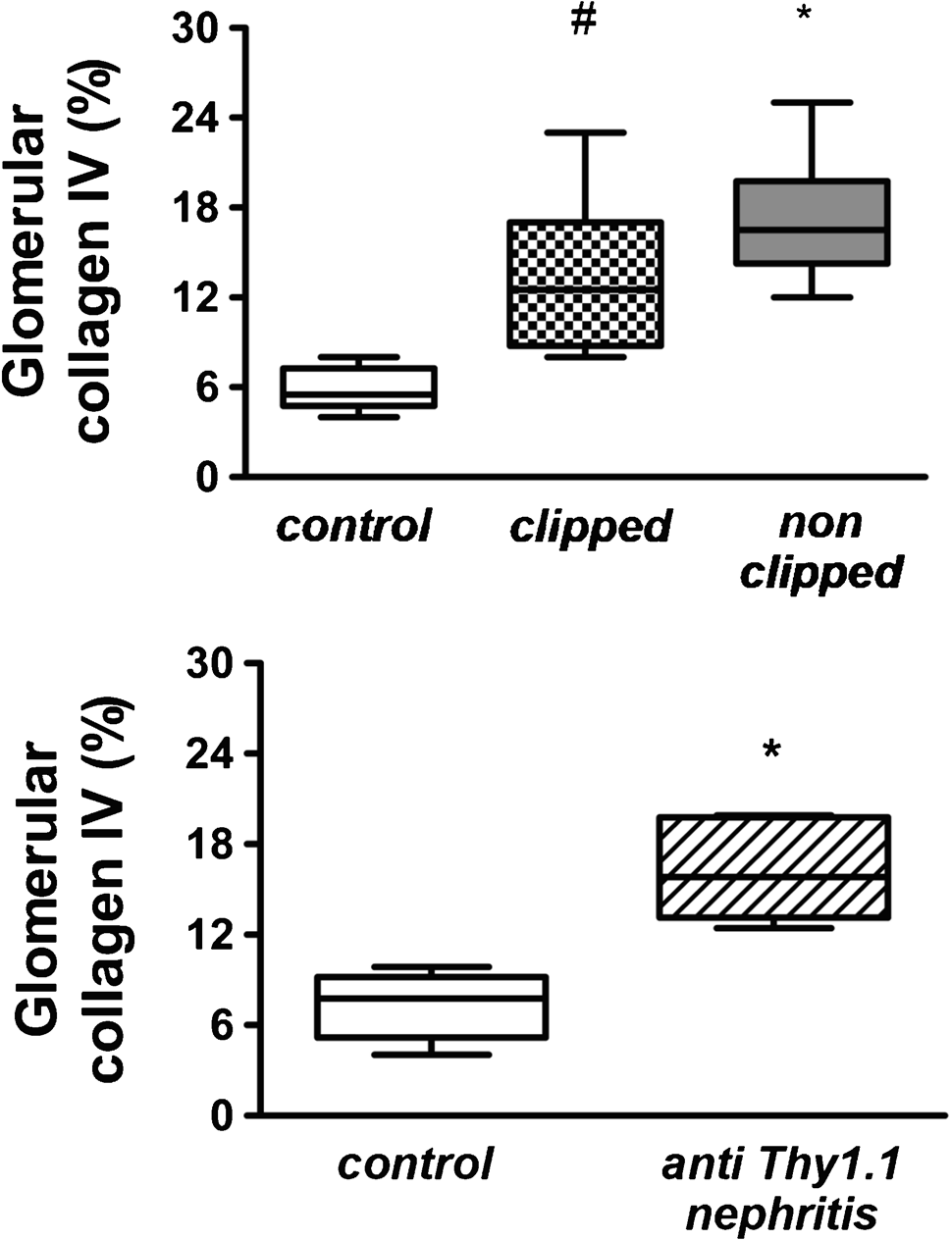
Upper panel: alteration of the fibrosis marker collagen IV in kidneys from 2K1C rats. In 2K1C rats, no significant difference between clipped and non-clipped kidneys occurred (control vs. clipped (#) vs. non-clipped (*). *n* = 6 per group, #*p* = 0.049 and *p = 0.001, normality test failed, ANOVA of ranks, all pairwise). Lower panel: increase of fibrosis marker in rats with anti-Thy1.1 nephritis (control vs. anti-Thy1.1 (*). *n* = 6 per group, *p = 0.001, normality test passed, one-way ANOVA, all pairwise)

Eventually, we assessed the pathological occurrence of renal collagen type IV as an indicator of fibrosis. The results in Fig. 5 show that collagen IV deposition was significantly increased as compared to normotensive controls but not different between the clipped and non-clipped kidneys. In mesangioproliferative GN, the collagen IV content was also significantly increased as compared to controls.

## Discussion

To our utmost surprise, our results completely contradicted our hypothesis that in experimental renovascular hypertension (2K1C), the excitability of afferent renal pathways is increased as assessed by the gain of tonic, highly active neurons: in samples of neurons with afferents from the clipped kidney, the portion of these tonic neurons decreased significantly instead, whereas the amount of phasic neurons increased as assessed by depolarizing current injection in vitro. We had also assumed that an alteration of neurons with renal afferents would correlate with the degree of renal inflammation. This proposition proved also to be wrong.

Our electrophysiological findings cannot be solely the consequence of some unspecific influences of the hypertensive model used since the number of tonic neurons with afferents from the clipped kidney increased dramatically after renal denervation, suggesting a specific influence emerging from the clipped kidney on renal afferent fibers and their first neurons in the dorsal root ganglia. In the experiments with high-pressure models, increases in afferent renal nerve activity have always been reported due to direct measurements or indirect experimental hints [2–4, 25, 33, 35, 36]. We had expected our results to reflect these reports by finding an increased number of tonic firing afferent neurons in our samples.

Neurons receiving afferents from the contralateral non-clipped kidney also yielded interesting and somewhat unexpected results: Neither in the distribution of highly active tonic to low active phasic neurons nor in the other characteristics any differences were found compared with neurons from controls. Obviously, neither the high blood pressure nor the ongoing inflammatory processes in these kidneys changed anything in the basic characteristics concerning firing patterns of the afferent neurons in the DRG.

Recently, in a nephritis model, we demonstrated that decreased afferent renal nerve activity also correlated with decreased numbers of tonic neurons with renal afferents [42]. Therefore, our unexpected results cannot be explained a priori by the fact that neurons in culture might be a model too artificial and thus ultimately unrelated to renal afferent innervation in vivo. The decrease of highly active tonic neurons in samples from animals with mesangioproliferative anti-Thy1.1 nephritis could again be confirmed in the present paper.

Ultimately, the interpretation of our extremely unexpected findings leads not so much to the question of whether sympathoexcitatory afferent neuronal pathways do exist but to the discussion about the extent to which likewise existing sympathoinhibitory reflex mechanisms from the kidney might occur, which under hypertensive conditions could lose their inhibitory influence, thus additionally favoring the development of high pathological sympathetic activity. From our experiments, we would infer at least that the loss of highly active tonic neurons in renovascular hypertension is not a completely marginal phenomenon.

In normal rats and pathological nonhypertensive models, sympathoinhibitory afferent reflex mechanisms have been described repeatedly. In the initial description of renal reflexes, an increase in activity of afferent nerve fibers on the ipsilateral side leads to renal sympathetic decreases on the contralateral side [28]. We demonstrated in previous experiments that afferent reflex mechanisms are likely to originate from the kidneys, which lead to sustained tonic sympathetic inhibition under control conditions [10]. In this study, stimulation of renal afferent neurons with capsaicin lead to long-lasting inhibition of RSNA, which could be reversed by blockade of substance P receptors (SP; NK-1R) in normotensive rats [10].

A high-salt diet remarkably induced an exacerbation of arterial hypertension in rats after selective destruction of afferent neuronal pathways. This phenomenon was not observed when afferent innervation remained intact [26]. Using mesangioproliferative nephritis as a model, we recently demonstrated that a decrease in afferent renal pathway activity contributed to poorer control of ultimately increased renal sympathetic activity [42]. This sympathoinhibitory effect of afferent renal nerves was strongly attenuated in Thy1.1 nephritis [41]. These findings indicate a loss of tonic sympathoinhibition rather than sympathoexcitation.

All these reports and the data from this in vitro study indicate the existence of sympatho-inhibitory reflex mechanisms via afferent nerve pathways, which are likely to be attenuated under certain pathological conditions. However, their role in sympathetic increases in hypertension has never been systematically investigated.

Based on the results of our earlier projects, we assumed that the reduction of highly active neurons in the samples of hypertensive animals must have something to do with the influence of renal afferents [41, 42].

In our opinion, this assumption was confirmed: 7 days after denervation of the clipped kidney, the number of these tonic renal neurons had more than normalized to a number above what was found in control samples. This strongly suggests that the sensory input from the clipped kidney impaired the availability of tonic neurons. These neurons are able to produce action potentials as expected under control conditions.

It has been reported that axotomy increased the excitability of sensory DRG neurons [13, 15, 20]. Furthermore, up to 25% of the investigated L5 DRG neurons developed a propensity to fire repetitively [20], which might be interpreted as tonic behavior. This is in accordance with our findings, even though renal T11-L2 DRG neurons [12] might not be directly comparable to L5 DRG neurons, but at least they might share some properties.

Although the renal afferent nerve fibers were axotomized, the corresponding renal DRG neurons (with afferent projections from the kidney) were still vital; otherwise, no such abundance of labeled tonic neurons could have been found.

It is tempting to speculate that after afferent renal denervation, an increased amount of tonic DRG neurons will increase some afferent (inhibitory) input due to their propensity to fire repetitively and spontaneously [20] after axotomy that was lost during renovascular hypertension.

The high blood pressure the non-clipped kidney is faced with was obviously not able to alter the firing pattern of afferent renal DRG neurons since the portion of tonic neurons with axons from the non-clipped kidney was similar compared to normotensive controls. Hence, altered mechanosensitivity of renal afferent nerves due to the increased arterial pressure in the non-clipped kidney might be not of major pathophysiological relevance. This is in accordance with earlier findings from rats with renovascular hypertension where mechanosensitive currents in DRG neurons with axons from the hind limb vasculature were increased, but no such increase was seen in DRG neurons with axons from the kidney [32]. It seems possible that renal autoregulation dampens the high blood pressure to a certain extent within the non-clipped kidney so that mechanosensitive qualities of renal afferent nerve fibers were less affected.

The clipped kidneys with renal artery stenosis showed a comparable degree of leucocyte infiltration and scarring as compared to the contralateral non-clipped kidney in the model of renovascular hypertension (2K1C) used. The immunohistochemical changes in mesangioproliferative glomerulonephritis were in accordance with our previous [49] findings.

We had expected that the extent of inflammation would be associated with changes in the stimulability of renal neurons in renovascular hypertension. We did not assume that the results would be in any case analogous to the situation in normotensive pathological models, when inflammation and fibrosis were rather favoring a reduction of tonic renal neurons, suggesting a decrease of afferent renal nerve activity. It is important to keep in mind that afferent nerve fibers of the autonomous nervous systems are not only chemosensitive but also mechanosensitive [27, 48], responding to a wide variety of stimuli [9, 17] in different situations [40], potentially resulting in stimulation or inhibition of afferent nerve fibers.

What we did not expect, however, is that in our renovascular hypertension model, the degree of inflammatory changes or fibrosis did not seem to have any readily detectable influence on neurogenic activity. In mesangioproliferative glomerulonephritis, the number of glomerular and interstitial macrophages as well as glomerular collagen IV deposition were significantly increased compared to controls as previously described [49]. If this is seen as a sign of inflammation, one could argue that the results obtained in renovascular hypertension will now exclude the possibility that in this form of glomerulonephritis, inflammation will influence afferent renal nerve fibers in spite of our results obtained from neuronal culture exposed to a cytokine such as CXCL1 [9]. This could be true but, in our eyes, it is as yet not possible to decide the matter in this way since we lack decisive observations as in the 2K1C model where we could compare results from two kidneys in one animal affected distinctively different by the disease model chosen.

Since inflammatory mediators are unlikely to have influenced the decreased number of tonic renal neurons in the rat model of renovascular hypertension, the following alternative explanations seem viable: afferent nerve fibers proved to be rather stimulated by sheer stress than by changes of the left ventricular end-diastolic pressure as we could demonstrate earlier in the series of experiments with a modified Langendorff heart setup [11]. The role of shear stress for the stimulation of afferent nerve fibers has been seen by others with respect to various sites of afferent autonomous innervation [7]. Since we investigated rats with renovascular hypertension in an earlier phase of the disease, renal autoregulation might have been able to considerably dampen blood pressure increases within the kidney. Furthermore, the hallmark of the pathophysiology of renovascular hypertension is a signal from the stenotic kidney that increases systemic blood pressure. Hence, the alteration seen in the neuronal sample of neurons with projections to the clipped kidney was quite likely. A drop in blood pressure will also decrease renal perfusion [45]. A consequence might have been in a stenotic kidney that altered intrarenal vascular shear stress [34] occurred, influencing the firing characteristics of neurons with renal afferents. It is not readily possible to assume a comparable explanation for the results in rats with mesangioproliferative nephritis since renal perfusion will likely not drop as low in nephritic kidneys as compared to a clipped kidney [45]. Hence, it is furthermore necessary to test the assumption that different pathomechanisms either depending on the mechanosensitivity or chemosensitivity of afferent renal nerve units are eventually necessary to explain the observation that in neuronal samples from rats both with renovascular hypertension and mesangioproliferative nephritis, the portion of tonic highly active neurons decreased. In this context, studies are also necessary to elucidate the role of biochemically distinguishable subgroups of neurons with afferent input from different renal compartments (e.g., pelvic vs. intrarenal) due to differential sensory input and the as yet undefined interaction of microglia, macrophages, and neurons along the afferent renal pathway [38].

Our results speak for a putatively complex multiplicity of afferent reflex mechanisms of the kidney that influence sympathetic activity. Further experiments, especially in vivo, will help to understand this diversity in more detail. This also seems important to more accurately understand and assess the effects and consequences of afferent renal nerve ablation in patients with hypertension.

Of note, the denervation of the clipped kidneys influenced significantly the frequency, the threshold voltage, and the action potential duration as compared to all other tonic groups of neurons (Table 3). Significant differences were furthermore observed between the tonic and phasic groups.

## Supplementary Information

Below is the link to the electronic supplementary material.

Supplementary file1 (PDF 731 KB)
