# Supplementary material for: Responsiveness of afferent renal nerve units in renovascular hypertension in rats
Source: Pflugers Arch. 2021 Jul 7;473(10):1617–29. doi: 10.1007/s00424-021-02591-6 (PMC8433106; doi:10.1007/s00424-021-02591-6)

Supplemental Material

Responsiveness of Afferent Renal Nerve Units in Renovascular Hypertension in Rats

Kristina Rodionova, Karl F. Hilgers, Salman Raffi-Fabrizii, Johannes Döllner, Nada Cordasic, Peter Linz, Anna-Lena Karl, Christian Ott, Roland E. Schmieder, Mario Schiffer, Kerstin Amann, Roland Veelken, Tilmann Ditting

Fig S1:

(a) *Representative sweep of action potentials from tonic renal neurons in control*

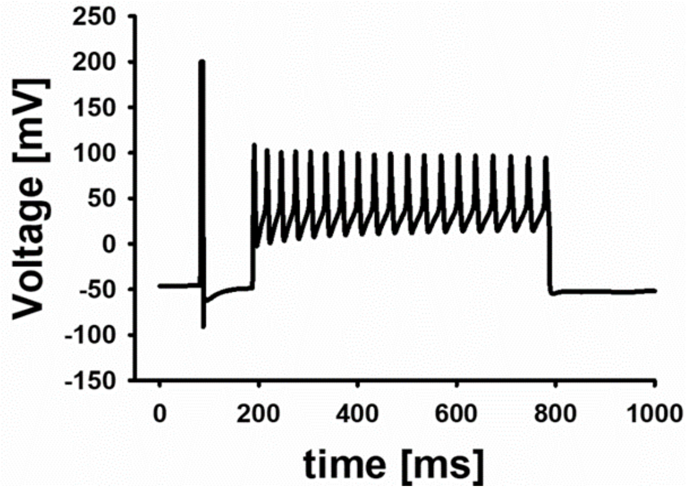

(b) *Representative sweep of action potentials from phasic renal neurons in controls*

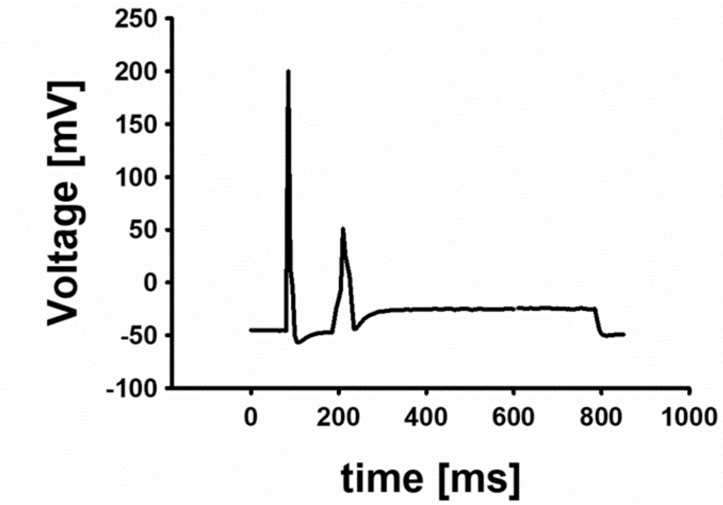

Fig S2:

(a) *Representative sweep of action potentials from tonic renal neurons in 2K1cl/clipped kidney*

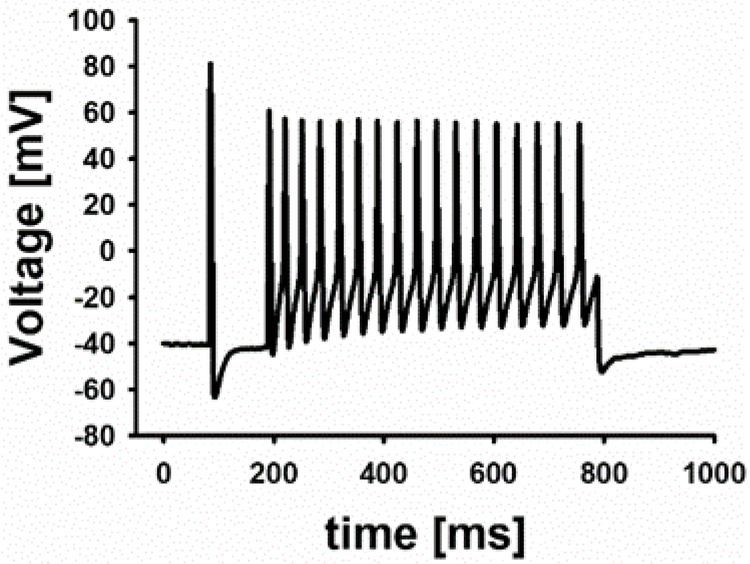

(b) *Representative sweep of action potentials from tonic neurons in controls 2K1cl/clipped kidney with denervation*

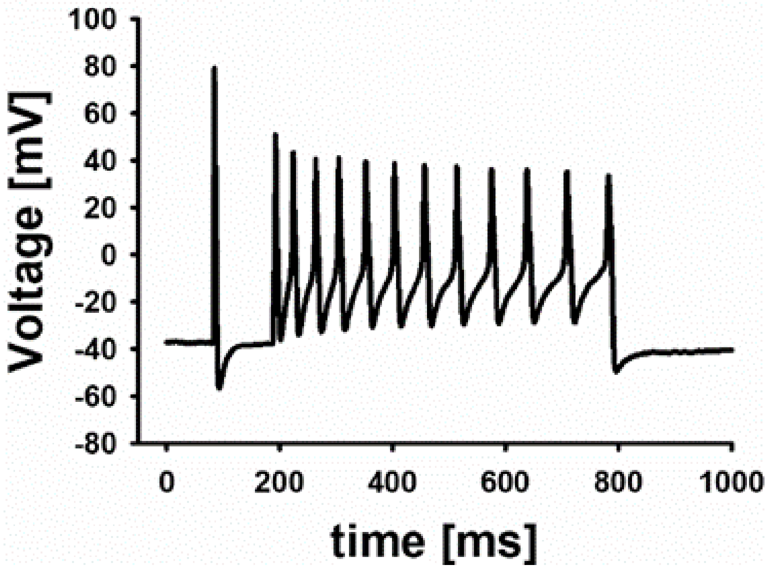

(c) *Representative sweep of action potentials from tonic neurons in controls 2K1cl/ non clipped kidney*

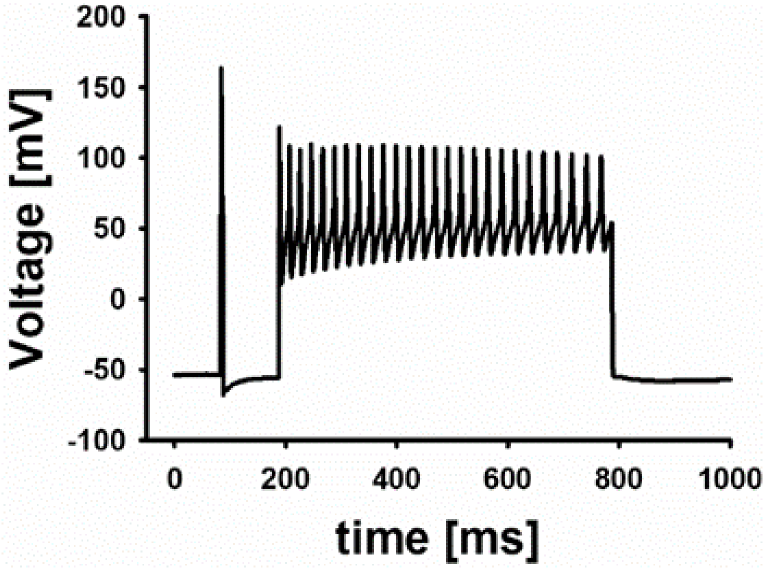

Fig S3:

Representative sweep of action potentials from tonic renal neurons in mesangio-proliferative nephritis

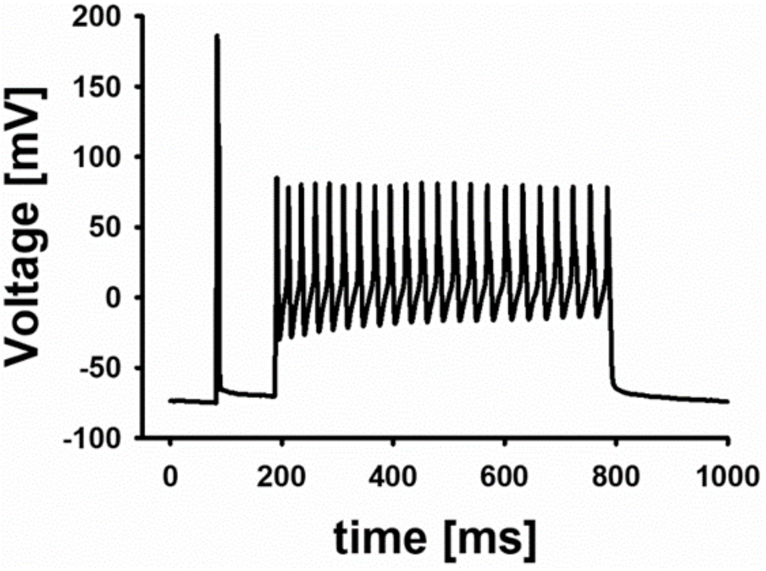

Fig S4:

Representative examples of renal inflammation analyzed by determination of macrophage infiltration into glomeruli and interstitium at the end of the experiment in rats with renovascular hypertension (3 weeks after implantation of a clip around one renal artery).

(a) control

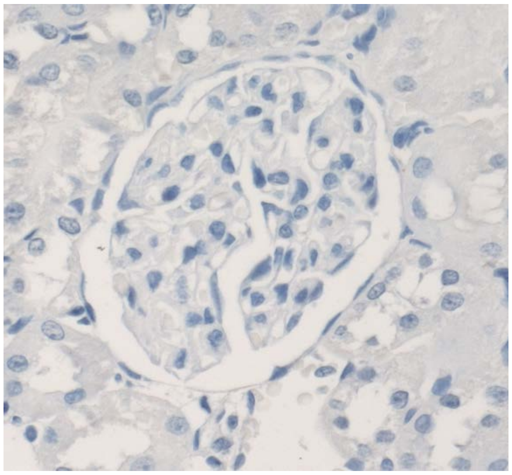

(b) clipped kidney

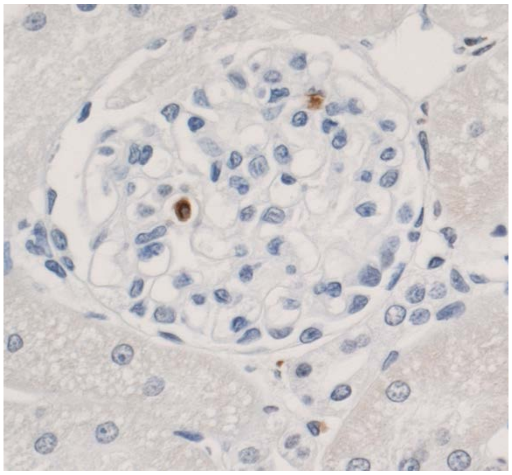

(c) non clipped kidney

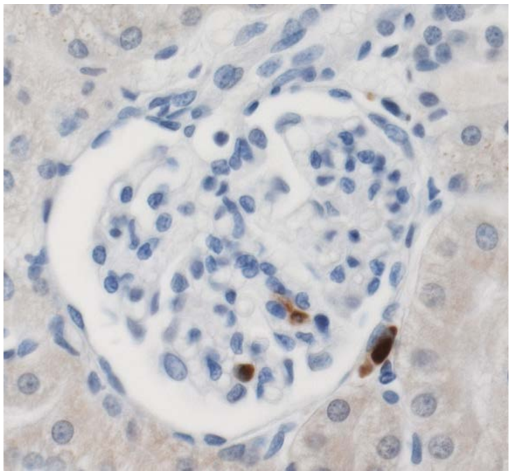

interstitial infiltration

(a) control

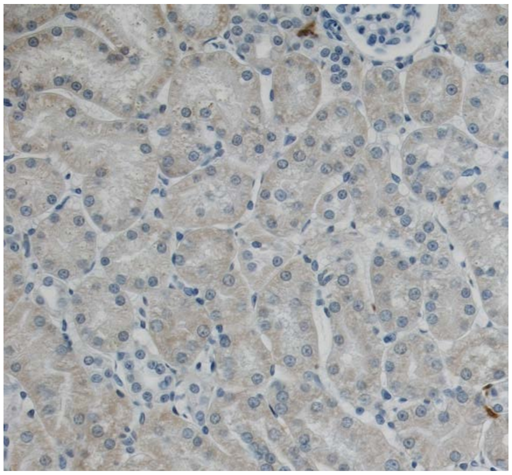

(b) clipped kidney

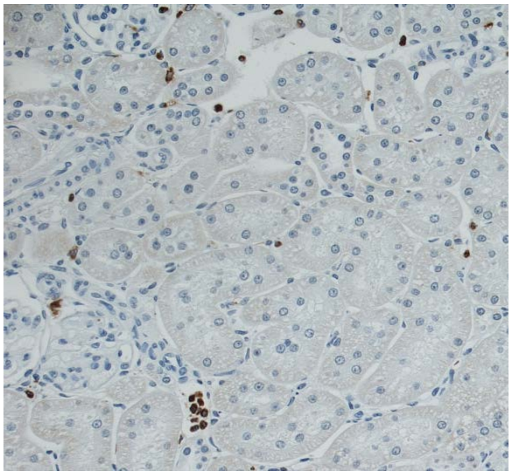

(c) non clipped kidney

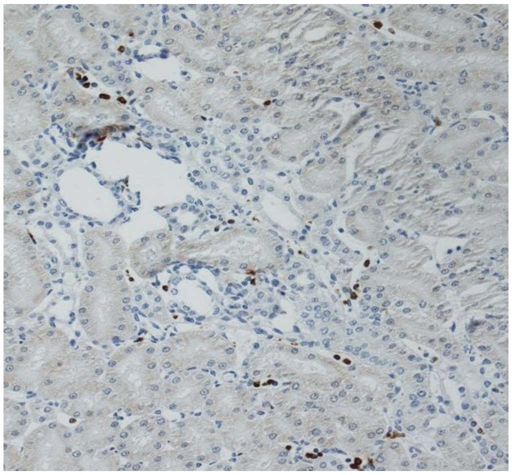

Fig S5:

Representative examples of renal inflammation analyzed by determination of macrophage infiltration into glomeruli and interstitium at the end of the experiment in rats with mesangioproliferative glomerulonephritis (6 d after anti–Thy-1.1 mAb injection).

<u>glomerular infiltration</u>

(a) control

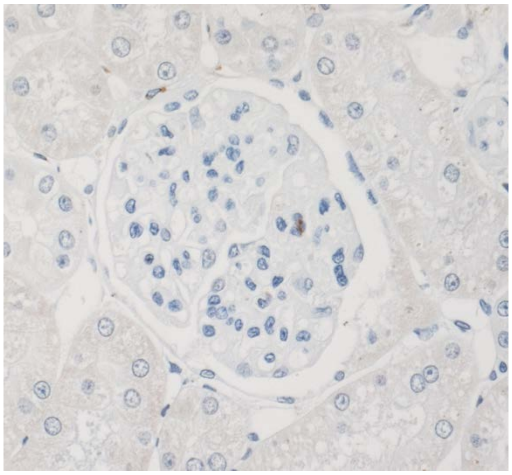

(b) mesangioproliferative nephritis

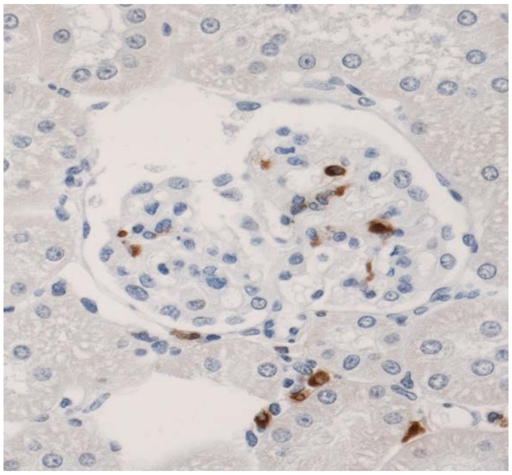

interstitial infiltration

(a) control

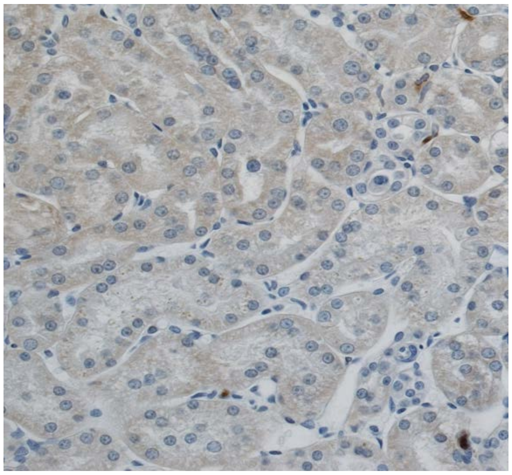

(b) mesangioproliferative nephritis

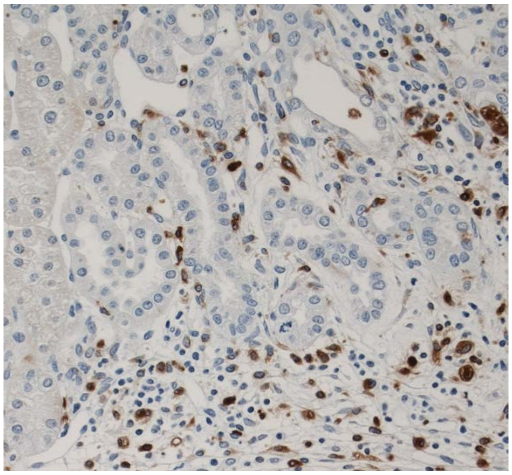

Supplement: Supplementary file 1 — Supplementary file1 (PDF 731 KB) [file 424_2021_2591_MOESM1_ESM.pdf]
